# SZT2 maintains hematopoietic stem cell homeostasis via nutrient-mediated mTORC1 regulation

**DOI:** 10.1172/JCI146272

**Published:** 2022-10-17

**Authors:** Na Yin, Gang Jin, Yuying Ma, Hanfei Zhao, Guangyue Zhang, Ming O. Li, Min Peng

**Affiliations:** 1 Department of Basic Medical Sciences, School of Medicine, and; 2 Institute for Immunology, Tsinghua University, Beijing, China.; 3 Tsinghua-Peking Center for Life Sciences, Beijing, China.; 4 Immunology Program, Sloan Kettering Institute, Memorial Sloan Kettering Cancer Center, New York, New York, USA.; 5 Immunology and Microbial Pathogenesis Program, Weill Cornell Graduate School of Medical Sciences, Cornell University, New York, New York, USA.; 6 Beijing Key Laboratory for Immunological Research on Chronic Diseases, Tsinghua University, Beijing, China.

**Keywords:** Hematology, Metabolism, Amino acid metabolism, Bone marrow transplantation, Hematopoietic stem cells

## Abstract

The mTORC1 pathway coordinates nutrient and growth factor signals to maintain organismal homeostasis. Whether nutrient signaling to mTORC1 regulates stem cell function remains unknown. Here, we show that SZT2 — a protein required for mTORC1 downregulation upon nutrient deprivation — is critical for hematopoietic stem cell (HSC) homeostasis. Ablation of SZT2 in HSCs decreased the reserve and impaired the repopulating capacity of HSCs. Furthermore, ablation of both SZT2 and TSC1 — 2 repressors of mTORC1 on the nutrient and growth factor arms, respectively — led to rapid HSC depletion, pancytopenia, and premature death of the mice. Mechanistically, loss of either SZT2 or TSC1 in HSCs led to only mild elevation of mTORC1 activity and reactive oxygen species (ROS) production. Loss of both SZT2 and TSC1, on the other hand, simultaneously produced a dramatic synergistic effect, with an approximately 10-fold increase of mTORC1 activity and approximately 100-fold increase of ROS production, which rapidly depleted HSCs. These data demonstrate a critical role of nutrient mTORC1 signaling in HSC homeostasis and uncover a strong synergistic effect between nutrient- and growth factor–mediated mTORC1 regulation in stem cells.

## Introduction

The mechanistic target of rapamycin complex 1 (mTORC1) kinase is a master regulator of anabolic cell growth and organismal homeostasis (1). Nutrients and growth factors are 2 major inputs for mTORC1 regulation (2). Two families of lysosome-residing small GTPases, RHEB and RAG, are responsible for relaying signals from growth factor and nutrient to mTORC1, respectively (3). Upstream of RHEB is the well-known PI3K/AKT pathway, which activates RHEB through phosphorylation of the tuberous sclerosis complex (TSC), a GTPase-activating protein (GAP) for RHEB (4, 5). In mammals, there are 4 RAG proteins, RagA– RagD, which form heterodimers to recruit mTORC1 to the lysosomal surface for activation by RHEB (6, 7). The GATOR1 complex inactivates RAGs during nutrient starvation by functioning as a GAP for RAGs (8). The lysosomal localization of GATOR1 is controlled by another protein complex called KICSTOR/SOG, and loss of its components, such as seizure threshold 2 (SZT2), results in nutrient–independent mTORC1 signaling (9, 10). Although the machinery involved in nutrient regulation of mTORC1 is becoming increasingly clear, the physiological significance remains largely unknown.

Balanced mTORC1 signaling is critical for maintenance of organismal homeostasis, and hyperactivation of mTORC1 is associated with multiple pathological conditions (1). Conventionally, constitutive mTORC1 signaling is studied in mice with loss-of-function PTEN or TSC1 mutations, which inactivates mTORC1 upon growth factor withdrawal or stress conditions (1). Nutrient deprivation–triggered mTORC1 inactivation is independent of PTEN and TSC1 but requires GATOR1 and KICSTOR (8–10). Germline knockout of key components of the GATOR1/KICSTOR complex, including DEPDC5, NPRL2, and SZT2, resulted in embryonic or neonatal lethality in mice (9, 11, 12), preventing investigation of the consequence of constitutive nutrient signaling to mTORC1 in adult mice.

Hematopoietic stem cells (HSCs) are responsible for hematopoiesis throughout adult life, and HSCs are highly sensitive to mTORC1 dysregulation (13). PTEN or TSC1 deficiency induces mTORC1 hyperactivation that leads to HSC exhaustion and leukemia in mice (14–17). The role of nutrient mTORC1 signaling in HSCs remains to be determined. In addition, the relationship between growth factor and nutrient regulation of mTORC1 in vivo remains unclear. In this study, we addressed these questions in HSCs.

## Results

### Steady-state hematopoiesis is largely preserved in the absence of SZT2.

We previously showed that SZT2 is essential for mTORC1 downregulation during nutrient deprivation, and mice with a germline deletion of SZT2 died quickly after birth due to defective mTORC1 downregulation during the neonatal fasting period (9). To study SZT2-mediated mTORC1 repression in adult mice, we generated a floxed allele of *Szt2* (Supplemental Figure 1A; supplemental material available online with this article; https://doi.org/10.1172/JCI146272DS1) and crossed *Szt2^fl/fl^* mice with mice expressing *Vav^Cre^_,_* which deletes genes in hematopoietic cells (18). PCR analysis of genomic DNA from total BM cells showed that *Szt2* was efficiently deleted (Supplemental Figure 1B).

The *Vav^Cre^Szt2^fl/fl^* mice (henceforth referred to as SZT2-KO mice) developed normally and did not show any gross abnormalities (data not shown). Consistent with previous studies showing that SZT2 negatively regulates mTORC1 (9, 10), SZT2-deficiency in hematopoietic lineages resulted in increased mTORC1 signaling in hematopoietic cells, including T cells, B cells, and myeloid cells, as well as hematopoietic stem and progenitor cells (Supplemental Figure 1, C and D). In agreement with mTORC1’s role in anabolic cell growth, hematopoietic cells from SZT2-KO mice had slightly increased cell size (Supplemental Figure 1, E–H). Together, these data demonstrate that SZT2 represses mTORC1 in hematopoietic lineages including HSCs.

The percentage and absolute number of lymphoid and myeloid cells in BM and spleen were comparable between WT and SZT2-KO mice at 6 weeks of age (Supplemental Figure 2, A and B). Age-dependent reduction in cellularity of spleen and BM from SZT2-KO mice was observed, which was mainly due to the reduction in number of B cells (Supplemental Figure 2, C and D). Similar findings were also reported in mice with hematopoietic deletion of TSC1 (19), indicating that B cells are particularly sensitive to mTORC1 hyperactivation.

Flow cytometric analysis of BM cells showed that immunophenotypic hematopoietic stem and progenitor cells, including Lin^–^c-Kit^+^Sca-I^+^ (LSK), Lin^–^c-Kit^+^Sca-I^–^ (MPP) and CD48^–^CD150^+^ LSK (LT-HSC) populations, were comparable between WT and SZT2-KO mice at 6 weeks of age (Supplemental Figure 3, A and B). At 15 weeks of age, SZT2-KO mice had an increased percentage of LSK cells, while MPP and LT-HSC numbers were unchanged (Supplemental Figure 3, C and D). These phenotypes were reminiscent of those observed in mice with an inducible deletion of PTEN or TSC1 at earlier time points (14–17). However, unlike mice with inducible deletions of PTEN or TSC1, which developed rapid HSC exhaustion and/or leukemia (14–17), SZT2-KO mice survived more than 1 year without obvious abnormalities (Supplemental Figure 3E).

### SZT2-deficiency dramatically decreases HSC reserves and repopulating potential.

**Figure 1:**
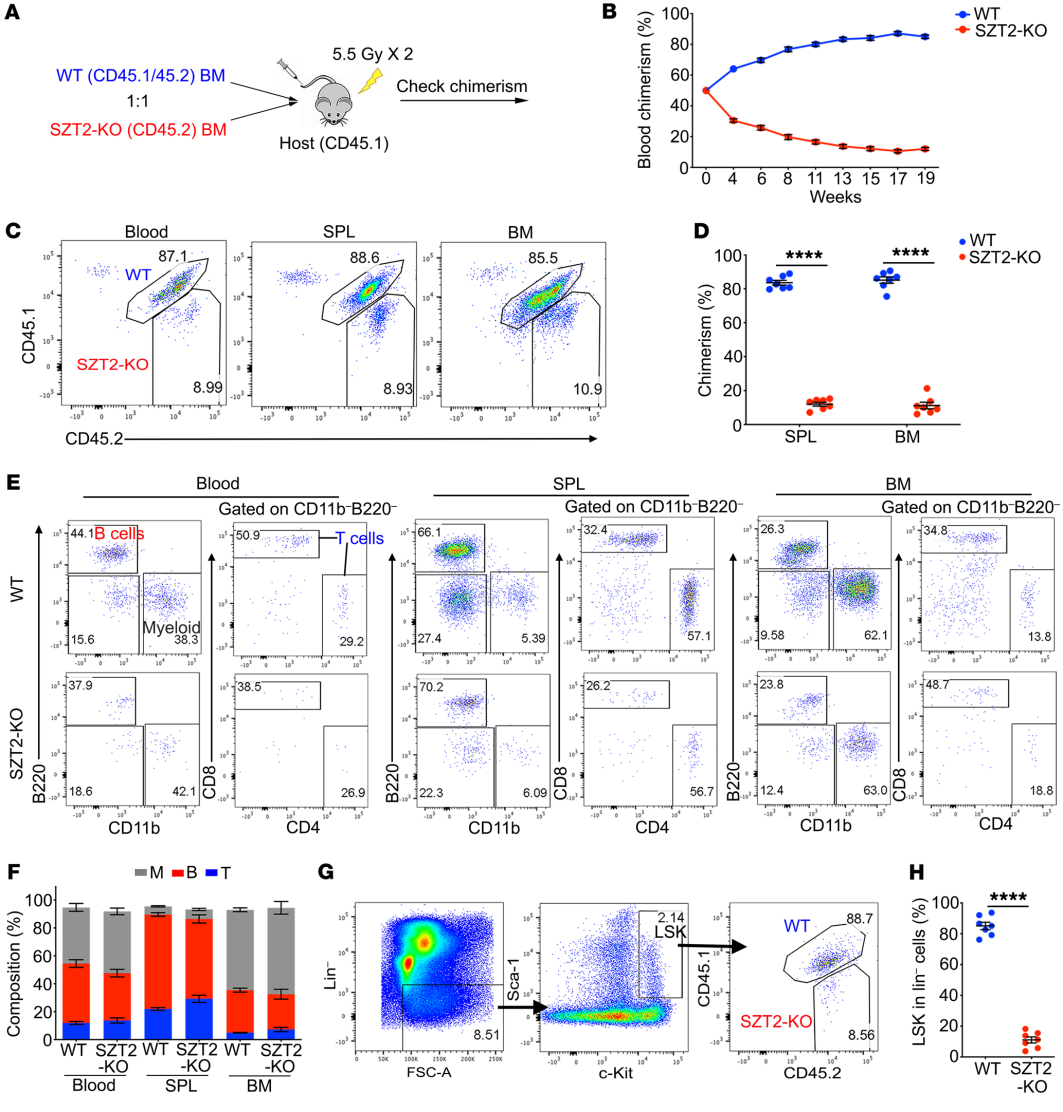
SZT2 is essential for the repopulating potential of HSCs. (**A**) A diagram of BM chimera experiment. WT (CD45.1/45.2) and *Vav^Cre^Szt2^fl/fl^* (SZT2-KO; CD45.2) BM cells were mixed at 1:1 ratio and injected into lethally irradiated CD45.1 recipient mice. Chimerism was monitored. (**B**) Blood chimerism of recipient mice at indicated time points. *n =* 7 mice per genotype, *P <* 0.0001 (2-way ANOVA). Data shown are representative of 4 independent experiments. (**C** and **D**) Chimerism of blood, spleen (SPL) and BM at 19 weeks after transplantation. Representative plots (**C**) and statistics (**D**) are shown. Data shown are representative of 2 independent experiments. (**E** and **F**) Frequencies of B cells (B220^+^), T cells (B220^–^CD11b^–^CD4^+^/CD8^+^) and myeloid cells (CD11b^+^) derived from WT or SZT2-KO BM. Representative plots (**E**) and summary data (**F**) are shown. (**G** and **H**) The contribution of WT and SZT2-KO BM to the Lin^–^c-Kit^+^Sca-I^+^ (LSK) population at 19 weeks after transplantation. Representative plots (**G**) and quantification (**H**) are shown. Data shown are representative of 2 independent experiments. In **D**, **F**, and **H**, data represent mean ± SEM, *n =* 7 mice per genotype, ****P < 0.0001 (paired 2-tailed *t* test.

BM transplantation (BMT) is the golden standard to test the self-renewal and repopulating potential of HSCs. To examine the ability of SZT2-deficient HSCs to repopulate irradiated hosts, we performed BM chimera experiments. Congenic, marked WT (CD45.1/45.2) and SZT2-KO (CD45.2) BM cells were mixed in a 1-to-1 ratio and injected into lethally irradiated mice (CD45.1) via tail vein, and chimerism was monitored from peripheral blood (Figure 1A). We found that the percentage of cells derived from SZT2-KO BM declined gradually in peripheral blood (Figure 1B). At 19 weeks after BMT, approximately 10% of cells in blood, spleen, and BM were from SZT2-KO donors (Figure 1, C and D). This was not due to different trafficking pattern between WT and SZT2-KO cells, since the percentages were almost identical among blood, spleen, and BM (Figure 1, B–D).

To explore whether the reduction of repopulating capacity of SZT2-KO HSCs is due to mTORC1 hyperactivation, we treated mice with the mTOR inhibitor rapamycin after BMT (Supplemental Figure 4A). In peripheral blood, the percentages of SZT2-KO BM-derived cells were higher in mice treated with rapamycin than those treated with the vehicle (Supplemental Figure 4B). Six weeks after BMT, the percentages of SZT2-KO BM-derived cells in spleen and lymphoid tissues were also higher in mice treated with rapamycin than those treated with vehicle (Supplemental Figure 4, C and D). These data demonstrate that the reduced repopulating capacity of SZT2-KO HSCs is at least partially due to mTORC1 hyperactivation.

Analysis of different lineages of cells in BM recipients showed that WT- and SZT2-KO BM-derived cells had similar percentages of T cells, B cells, and myeloid cells (Figure 1, E and F), indicating that SZT2-deficiency does not cause biased hematopoiesis. In BM, LSK populations derived from SZT2-KO donors were significantly reduced (Figure 1, G and H), indicating that the reduced repopulating potential of SZT2-deficient BM was at the stem/progenitor cell level. Taken together, these data demonstrate that repression of mTORC1 activation by SZT2 is critical for the self-renewal and repopulating potential of HSCs.

### Ablation of both SZT2 and TSC1 results in rapid HSC exhaustion, pancytopenia, and premature death of mice in a mTORC1-dependent manner.

SZT2 and TSC1 repress mTORC1 in response to different signals. TSC1 inactivates mTORC1 upon growth factor deprivation or stress conditions (4, 5), while SZT2 represses mTORC1 upon nutrient starvation (9, 10). To examine the relationship between TSC1- and SZT2-mediated mTORC1 inactivation, we crossed SZT2-KO mice with *Tsc1^fl/fl^* mice (20) to generate *Vav^Cre^Szt2^fl/fl^Tsc1^fl/fl^* mice (henceforth referred to as DKO mice). As a control, we also deleted TSC1 with *Vav^Cre^* (*Vav^Cre^Tsc1^fl/fl^* mice [henceforth referred to as TSC1-KO mice]) for side-by-side comparison. In addition, to exclude any functions of TSC1 or SZT2 that are not mTORC1-related but could complicate the interpretation of the phenotype of DKO mice, we further deleted the mTORC1-defining component RAPTOR in DKO mice. We crossed DKO mice with *Rptor^fl/fl^* mice (21) to generate *Vav^Cre^Szt2^fl/fl^ Tsc1^fl/fl^Rptor^fl/fl^* mice (designated as triple knockout [henceforth referred to as TKO mice]). Thus, if a phenotype of DKO mice was not rescued by RAPTOR-deficiency in TKO mice, then the phenotype was not caused by mTORC1 hyperactivation.

**Figure 2:**
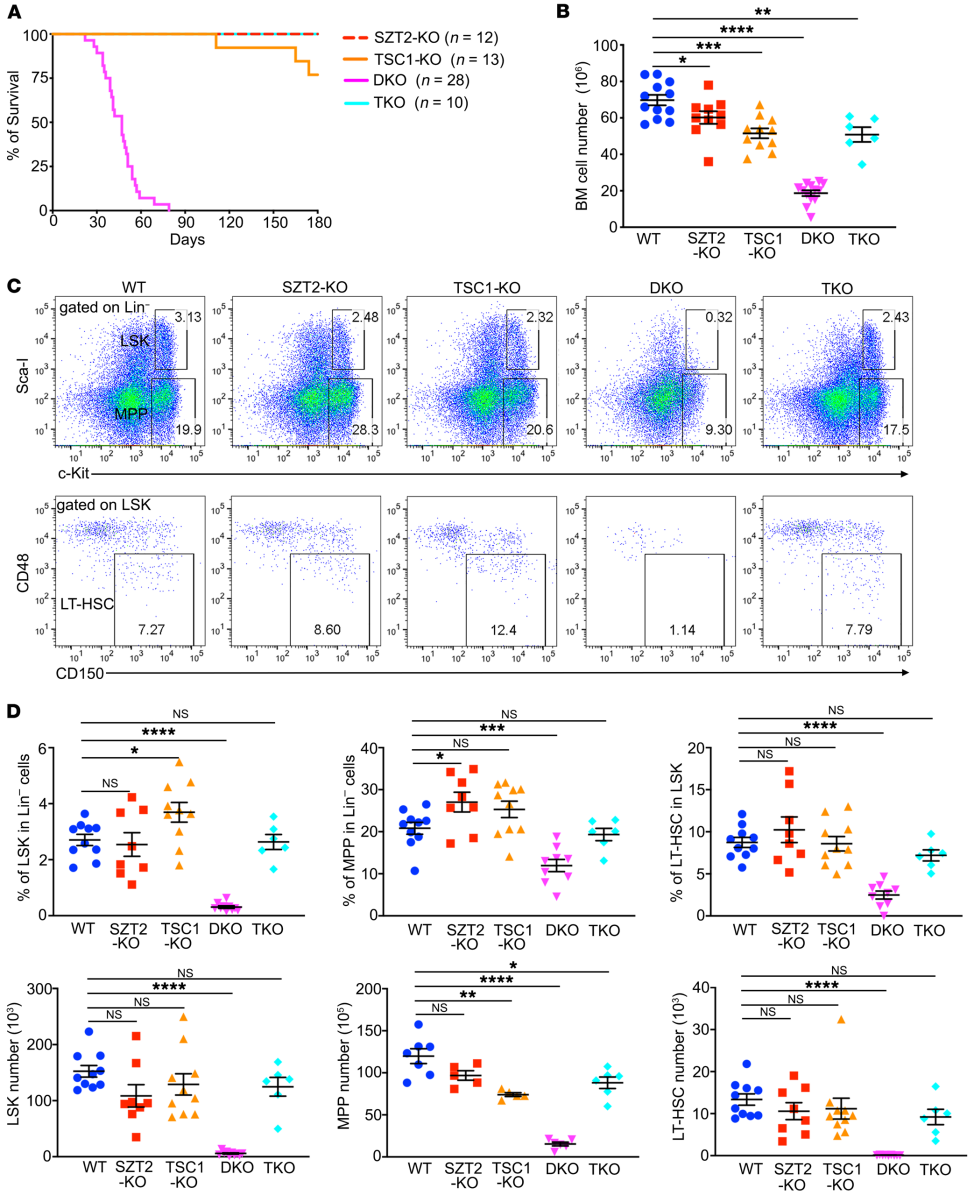
Loss of both SZT2 and TSC1 results in rapid HSC depletion, pancytopenia and early lethality of mice in a mTORC1-dependent manner. (**A**) Survival curves of *Vav^Cre^Szt2^fl/fl^* (SZT2-KO), *Vav^Cre^Tsc1^fl/fl^* (TSC1-KO), *Vav^Cre^Szt2^fl/fl^ ^fl^Tsc^1fl/fl^* (DKO) and *Vav^Cre^Szt2^fl/fl^ Tsc1f^l/fl^Rptor^fl/fl^* (TKO) mice. (**B**) Total cell numbers of BM from mice with indicated genotypes at 4 weeks old, *n =* 6–12 mice per genotype. (**C** and **D**) Flow cytometry analysis of frequencies of Lin^–^c-Kit^+^Sca-I^+^ (LSK), Lin^–^c-Kit^+^Sca-I^–^ (MPP), and CD48^–^CD150^+^ LSK (LT-HSC) populations in BM of mice with indicated genotypes. Representative plots (**C**) and quantification (**D**) are shown. Data shown are representative of 3 independent experiments. In **B** and **D**, data represent mean ± SEM, 1-way ANOVA followed by Dunnett’s test, *P < 0.05, **P < 0.01, ***P < 0.001, ****P < 0.0001.

Strikingly, when SZT2 and TSC1 were both deleted in hematopoietic lineage cells (Supplemental Figure 5, A and B), the resultant DKO mice died prematurely starting at 2 weeks of age, and all DKO mice succumbed to death by 11 weeks of age, with a median survival of 5 weeks (Figure 2A). Previous studies have shown that inducible deletion of TSC1 in HSCs resulted in defective hematopoiesis (16, 17). We found that constitutive deletion of TSC1 with *Vav^Cre^* did not significantly affect hematopoiesis at young ages, and most mice survived more than 6 months (Figure 2A), which is consistent with a recent report (19). The premature-death phenotype of DKO mice was not observed in TKO mice (Figure 2A), demonstrating that the phenotype is dependent on mTORC1 hyperactivation.

Analysis of BM cellularity at 4 weeks of age showed that both SZT2-KO and TSC1-KO mice had slightly reduced cell numbers, while DKO mice had a significant reduction in cellularity, which was rescued in TKO mice (Figure 2B). Flow cytometric analysis of BM cells showed that LSK and MPP populations were largely preserved in SZT2-KO mice and TSC1-KO mice, were almost absent in DKO mice, and were rescued in TKO mice (Figure 2, C and D). Together, these data demonstrate that TSC1 and SZT2 have a strong synergistic effect on the homeostasis of HSCs, and loss of both proteins results in HSC depletion and BM failure in a mTORC1-dependent manner.

### The hematopoietic niche is intact in the absence of SZT2 and TSC1.

**Figure 3:**
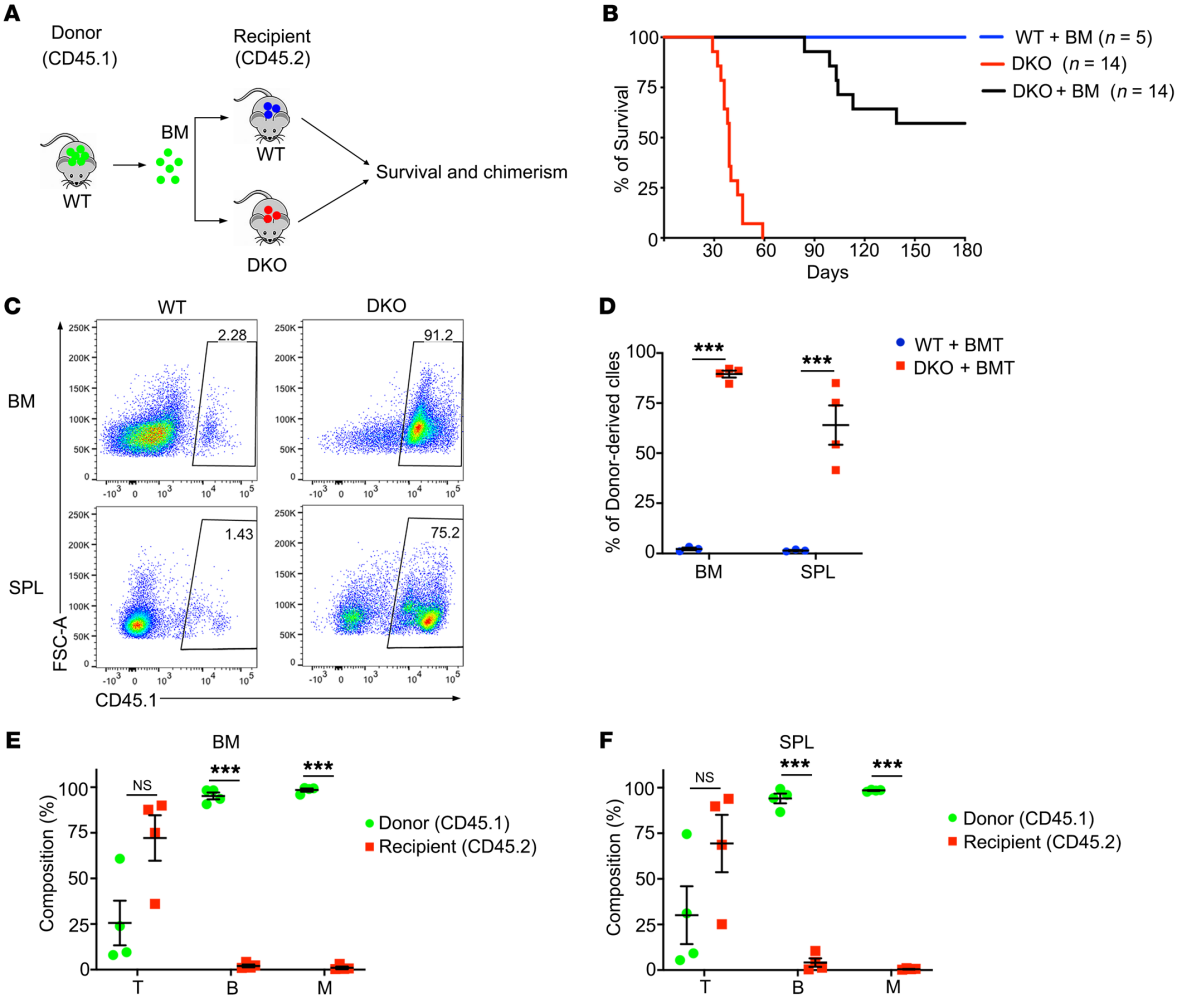
The hematopoietic niche is intact in the absence of SZT2 and TSC1. (**A**) A diagram of BM transplantation. Total BM cells from WT (CD45.1) mice were retroorbitally injected into nonirradiated WT (CD45.2) or *Vav^Cre^Szt2^fl/fl^ ^fl^Tsc^1fl/fl^* (DKO) (CD45.2) mice. Survival and chimerism were monitored. (**B**) Survival curves of mice with indicated treatment. (**C** and **D**) Percentages of donor cells (CD45.1^+^ ) in BM of WT and DKO recipients at 5 weeks after transplantation. (**C**) Representative plots and (**D**) quantification are shown. Data shown are representative of 2 independent experiments. (**E** and **F**) Frequencies of B cells (B220^+^), T cells (B220^–^CD11b^–^CD3e^+^) and myeloid cells (CD11b^+^) in BM (**E**) or spleen (SPL) (**F**) from the DKO recipient mice. Data shown are representative of 2 independent experiments. In **D**, **E**, and **F**, data represent mean ± SEM, *n =* 4 mice per genotype, unpaired 2-tailed *t* test, ***P < 0.001.

Although *Vav^Cre^* is mainly expressed in hematopoietic cells, studies have shown that it can delete genes in nonhematopoietic cells, including endothelial cells and bone cells that form niches for HSCs (22). To investigate whether the phenotype of DKO mice described above is intrinsic to HSCs, we performed a BMT experiment to rescue the premature death of DKO mice. We retroorbitally injected WT BM cells into WT and DKO mice on P7 when DKO mice were still healthy (Figure 3A). About half of the DKO mice were rescued from premature death (Figure 3B), indicating that the BM niche of DKO mice is intact and able to support hematopoiesis.

Flow cytometric analysis of rescued DKO mice showed that donor BM cells successfully engrafted in DKO, but not WT, mice without irradiation (Figure 3, C and D), further supporting that HSCs were depleted in these mice, creating an empty niche. Although both B cells and myeloid cells were completely derived from donor BM cells, some T cells in DKO mice were still from the host (Figure 3, E and F), suggesting that T cells are relatively resistant to mTORC1 hyperactivation.

### SZT2 and TSC1 synergistically repress mTORC1 activity and ROS production in HSCs.

**Figure 4:**
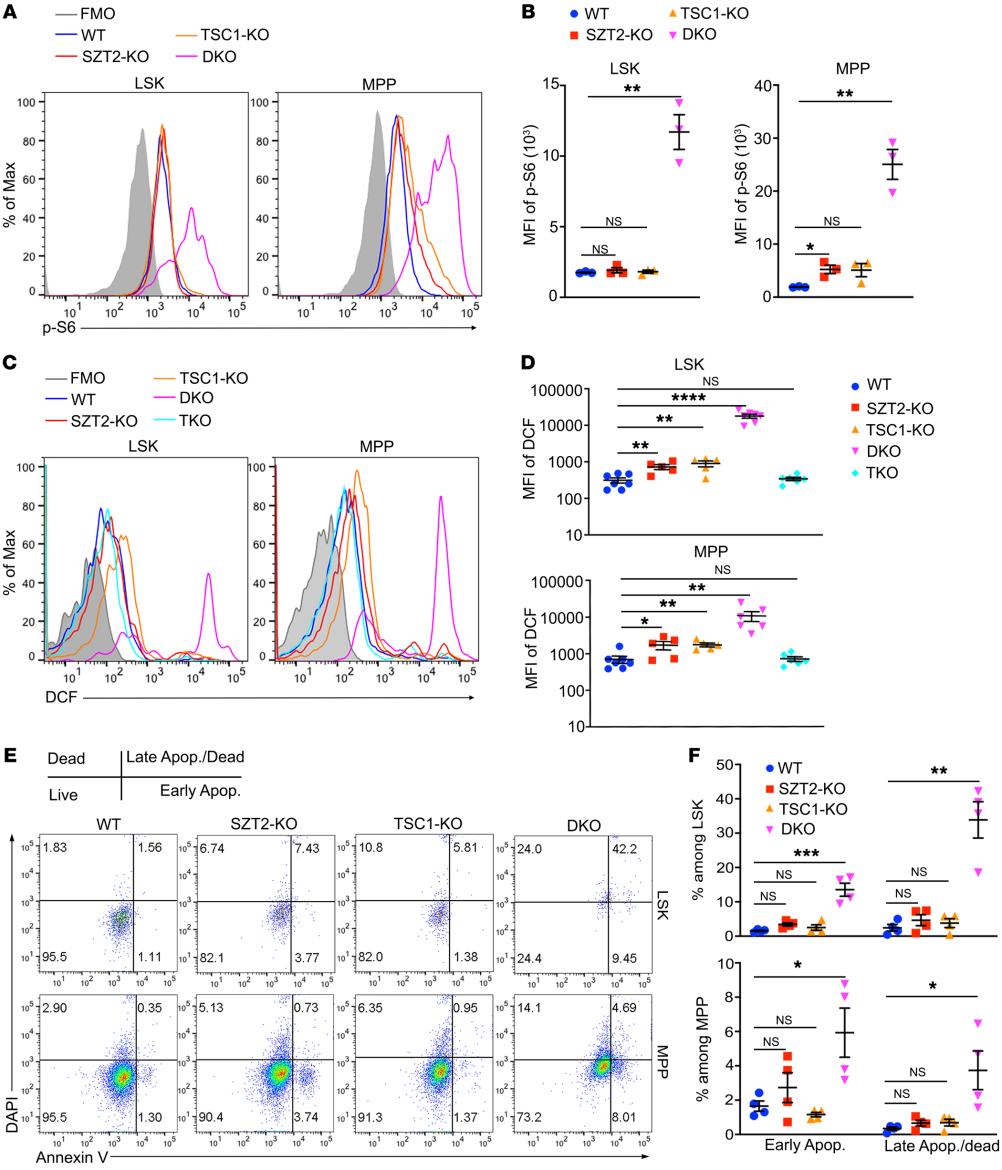
SZT2 and TSC1 synergistically repress mTORC1 activation and ROS production in HSCs. (**A** and **B**) Phosphorylation of ribosomal S6 protein (pS6) in Lin^–^c-Kit^+^Sca-I^+^ (LSK) and Lin^–^c-Kit^+^Sca-I^–^ (MPP) populations from WT, *Vav^Cre^Szt2^fl/fl^* (SZT2-KO), *Vav^Cre^Tsc1^fl/fl^* (TSC1-KO), or *Vav^Cre^Szt2^fl/fl^ ^fl^Tsc^1fl/fl^* (DKO) mice at 4 weeks old was measured by flow cytometry. Representative plots (**A**) and statistics of mean fluorescence intensity (MFI) (**B**) are shown, *n =* 3 mice per genotype. Data shown are representative of 2 independent experiments. (**C** and **D**) Flow cytometry analysis of ROS level in LSK and MPP populations from mice with indicated genotypes at 4 weeks old. Representative plots (**C**) and statistics (**D**) are shown, *n =* 5–7 mice per genotype. Data shown are representative of 2 independent experiments. (**E** and **F**) Flow cytometry analysis of apoptosis of LSK and MPP populations from mice with indicated genotypes at 4 weeks old. Representative plots (**E**) and statistics (**F**) are shown, *n =* 4 mice per genotype. Data shown are representative of 2 independent experiments. In **B**, **D**, and **F**, data represent mean ± SEM, 1-way ANOVA followed by Dunnett’s test, *P < 0.05, **P < 0.01, ****P < 0.0001.

To explore the mechanism of synergistic effect between SZT2 and TSC1, we first analyzed mTORC1 activity. Due to the early death of the DKO mice, we performed analysis with mice at ages of 3–4 weeks. At this age, the activity of mTORC1 in LSK cells was unchanged in SZT2-KO or TSC1-KO mice, while mTORC1 in MPP cells was slightly increased (Figure 4, A and B). However, mTORC1 activity in LSK and MPP cells was dramatically increased in DKO mice (approximately 10-fold) (Figure 4, A and B), which was reversed to WT level by RAPTOR deficiency in TKO mice (Supplemental Figure 5, C and D). These data demonstrate that either TSC1 or SZT2 can keep mTORC1 in check at this age, while loss of both proteins results in full mTORC1 activation, which requires RAPTOR, the defining component of mTORC1. In addition, the enlarged cell size of LSK and MPP cells from DKO mice was reversed to WT level in TKO mice (Supplemental Figure 5, E and F), demonstrating that the bigger cell size of the cells in the DKO mice is caused by mTORC1 hyperactivation. Finally, the pancytopenia observed in DKO mice was fully rescued by RAPTOR deficiency in TKO mice (Supplemental Figure 6), again demonstrating that phenotypes in DKO mice are due to mTORC1 hyperactivation.

Increased mTORC1 signaling has been shown to boost ROS production in HSCs (17). We found that loss of either TSC1 or SZT2 caused a slight increase of ROS production in LSK and MPP cell populations, while loss of both TSC1 and SZT2 in DKO mice resulted in approximately a 100-fold increase of ROS (Figure 4, C and D). This dramatic increase of ROS in DKO mice was reversed to WT level in TKO mice (Figure 4, E and F), demonstrating that ROS production in DKO mice is due to mTORC1 hyperactivation. ROS is detrimental to quiescent stem cells (13). Indeed, while apoptosis of LSK and MPP cells in SZT2-KO or TSC1-KO mice was comparable to that of WT mice, apoptosis of these cells was dramatically increased in DKO mice (Figure 4, E and F), which was rescued by RAPTOR-deficiency in TKO mice (Supplemental Figure 5, G and H). Together, these data demonstrate that SZT2 and TSC1 synergistically repress mTORC1 activation in HSCs and that loss of both proteins results in superactivation of mTORC1, leading to fulminant ROS production and HSC apoptosis.

## Discussion

Our studies demonstrated that constitutive nutrient signaling to mTORC1 in the absence of SZT2 was detrimental to the repopulating capacity of the HSCs. A previous study investigated the consequence of constitutive–nutrient mTORC1 signaling in HSCs using RagA^GTP/GTP^ mice (23), which concluded that the nutrient signaling does not affect HSC function (24). An explanation for the discrepancy is that donor cells from that study were from fetal liver (due to the postnatal lethality of RagA^GTP/GTP^ mice), while in our study we used BM cells from adult mice. Additionally, the RagA^GTP/GTP^ point mutation (D66L) knockin mice had a significant reduction of RagA protein levels (23), thus, mTORC1 activity in this strain may be lower than that of SZT2-deficient mice.

mTORC1 hyperactivation has been studied in mice with TSC1 deficiency in numerous studies and in various organs and tissues; here, we provide a different model of constitutive mTORC1 signaling with SZT2 deficiency. The phenotype of SZT2 deficiency in HSCs is milder than that of TSC1 deficiency or PTEN deficiency. We believe that this is, in fact, an advantage of this model. SZT2 specifically represses mTORC1 during fasting, thus, SZT2-deficiency mimics overnutrition-induced mTORC1 elevation, which is a chronic condition associated with common human diseases, including obesity, diabetes, cancer, and other aging-related diseases (1). Thus, SZT2-deficiency is an ideal model to dissect the relationship among overnutrition, mTORC1 hyperactivation, and disease.

Nutrients and growth factors are 2 major inputs for the mTORC1 pathway, but the relationship between nutrient- and growth factor–mediated mTORC1 regulation in vivo was unclear. Our data demonstrate that, as key repressors of mTORC1 on nutrient and growth factor arms, respectively, SZT2 and TSC1 do not simply work in an additive manner, but instead show a dramatic synergistic effect. This suggests that SZT2 and TSC1 function as fail-safe mechanisms for each other in order to keep mTORC1 from overactivation. The extremely high level of ROS observed in HSCs devoid of both SZT2 and TSC1, but not either protein alone, is still mTORC1-dependent, demonstrating that mTORC1 is a key driver of ROS production in HSCs. How overactivation of mTORC1 induces such high levels of ROS in DKO HSCs warrants further investigation. In addition, whether ROS production is the major driver of HSC apoptosis in DKO mice requires further investigation as well. SZT2 was initially identified in mice with epilepsy, and recurrent mutations of SZT2 are also reported in patients with epilepsy (25). Whether these patients also have defects in their hematopoietic systems is of interest.

## Methods

### Mice.

*Szt2^fl/+^* mice (Szt2^tm1a(KOMP)Wtsi^), in which exons 8 and 9 were flanked by 2 loxp sites (Supplemental Figure 1A), were obtained from EUCOMM. The mice were first crossed with a transgenic Flipase strain (The Jackson Laboratory, stock no. 005703) to remove the Neo cassette, and then crossed with *Vav^Cre^*-transgenic mice (The Jackson Laboratory, stock no. 008610) to delete *Szt2* in hematopoietic cells. B6.SJL (CD45.1, stock no. 002014), *Tsc1^fl/fl^* (stock no. 005680), and *Rptor^fl/fl^* (stock no. 013188) mice were also from The Jackson Laboratory. Mice were housed under specific pathogen–free conditions at the Laboratory Animal Research Center of Tsinghua University. Both male and female mice were used in experiments.

### Antibodies.

Fluorescent-dye-labeled antibodies against CD45.1 (clone A20; BioLegend), CD45.2 (clone 104; eBioscience), CD4 (clone GK1.5; BioLegend), CD8 (clone 53.6.7; BioLegend), CD11b (clone M/170; eBioscience), TER119 (clone TER-119; BioLegend), B220 (clone 6D5; BioLegend), CD3 (clone 145-2C11; BioLegend), Gr1 (clone RB6-RC5; BioLegend), CD41 (clone MWReg30; BioLegend), CD48 (clone HM48-1; BioLegend), CD150 (clone TC15-12F12.2; BioLegend), c-Kit (clone 2B8; BioLegend), and Sca-I(clone D7; eBioscience) were purchased from eBioscience or BioLegend. Phospho-S6 (Ser235/236, clone D57.2.2E) Alexa Fluor 647 was purchased from CST. Blocking antibody for Fc**γ**R (clone 2.4G2) was obtained from Bio X Cell. Raptor (clone E6O3A) rabbit mAb and TSC1 (clone D43E2) rabbit mAb used in immunoblot were from CST.

### Flow cytometry.

Cells from bone marrow and spleen were depleted of erythrocytes by hypotonic lysis. Cells were incubated with specific antibodies for 15 minutes on ice in the presence of anti-Fc**γ**R to block Fc**γ**R binding. Dead cells were excluded by DAPI (Invitrogen) staining. HSC compartments (HSC, Lin^–^c-Kit^+^Scar1^+^ and MPP, Lin^–^c-Kit^+^Scar1^–^) were identified by flow cytometry using the antibodies against c-Kit and Sca1, along with the following antibodies against lineage markers, TER119, B220, Gr1, CD11b, CD3, CD4, and CD8. Long-term HSCs (LT-HSC, Lin^–^c-Kit^+^Scar1^+^CD150^+^CD48^–^) were further identified by antibodies against CD150 and CD48. For phospho-S6 staining, BM cells were flushed out by 1% PFA, fixed for 10 minutes at room temperature (RT) and then stained with HSC surface markers at 4°C for 10 minutes after washing twice with PBS. Cells were fixed for 20 minutes at RT in transcription factor (TF) Fixation/permeabilization buffer (BD Bioscience), washed twice with PermWash buffer (BD Bioscience), and incubated for 1 hour at RT with the Alexa Fluor 647–conjugated antibody against phospho-S6 ribosomal protein (Ser235/236) (D57.2.2E, CST) for 1 hour at RT. All samples were acquired with an Fortessa flow cytometer (BD) and analyzed with FlowJo software (TreeStar).

### Bone marrow transplantation.

BM cells were extracted from donor *Vav^Cre^Szt2^fl/fl^* (CD45.2) or WT (CD45.1/45.2) mice and subjected to Ficoll-Paque Plus (GE Healthcare) centrifugation to get rid of granule and erythrocyte cells. Recipient (CD45.1 WT) mice were lethally irradiated (4.5 Gy twice, separated by 2 hours). A 1:1 mixture of CD45.2 donor (*Vav^Cre^Szt2^fl/fl^*) and CD45.1/45.2 WT competitor BM cells were prepared in PBS and injected into recipient mice via tail vein. For each recipient, a total of 1 million nucleated bone marrow cells were injected. Peripheral blood was sampled and analyzed every 2 to 3 weeks by flow cytometry. To rescue the early lethality of DKO mice, total BM cells from CD45.1 mice were retroorbitally injected into DKO mice on P7. Survival and blood chimerism were monitored. For the rapamycin rescue experiment, recipient mice were treated with rapamycin (2 mg/kg, intraperitoneal injection) or vehicle every 2 days for 6 weeks.

### ROS measurement.

Freshly isolated BM cells were stained with surface marker on ice and then washed in PBS. Cells were incubated for 10 minutes at 37°C with CM-H2DCF-DA (Thermo Fisher Scientific). Cells were then analyzed by flow cytometry after returning the cells to prewarmed growth medium.

### Apoptosis measurement.

Freshly isolated BM cells were stained with surface marker on ice and then washed in PBS. Cells were resuspended in binding buffer (eBioscience) and incubated with Annexin V-FITC (eBioscience) for 10 minutes at RT, washed, and Propidium Iodide (BioLegend) was added. Cell apoptosis and death were analyzed by flow cytometry.

### Statistics.

Statistical tests were performed with Prism (GraphPad). They include 1- and 2-way ANOVA, paired and unpaired 2-tailed *t* tests, and Dunnett’s test. A value of *P <* 0.05 was considered significant. All error bars represent SEM.

### Study approval.

All animal work was approved by IACUC of Tsinghua University. Mice were housed under specific pathogen–free conditions at the Laboratory Animal Research Center of Tsinghua University. These animal facilities were approved by Beijing Administration Office of Laboratory Animals.

## Author contributions

NY and MP conceived the study. NY, GJ, and MP planned and performed experiments, analyzed data, and wrote the manuscript. YM performed experiments. HZ and GZ provided help with experiments. MOL provided key mouse lines utilized in this study. MP supervised the study. All authors read and approved the manuscript.

## Supplementary Material

Supplemental data

## Version 1 10/17/2022

Electronic publication
