# Supplementary material for: SZT2 maintains hematopoietic stem cell homeostasis via nutrient-mediated mTORC1 regulation
Source: J Clin Invest. 2022 Oct 17;132(20):e146272. doi: 10.1172/JCI146272 (PMC9566891; doi:10.1172/JCI146272)
Supplement: Supplemental data [file jci-132-146272-s094.pdf]

Supplementary material for

SZT2 maintains hematopoietic stem cell homeostasis via nutrient-mediated mTORC1 regulation

Na Yin[1,2*], Gang Jin[1,2*], Yuying Ma[1,2], Hanfei Zhao[1,2], Guangyue Zhang[1,2,3], Ming O. Li[5,6], Min Peng[1,2,3,4]

Affiliations:

[1]Department of Basic Medical Sciences, School of Medicine, Tsinghua University, Beijing 100084, China

[2]Institute for Immunology, Tsinghua University, Beijing 100084, China

[3]Tsinghua-Peking Center for Life Sciences, Beijing 100084, China

[4]Beijing Key Laboratory for Immunological Research on Chronic Diseases, Tsinghua University, Beijing 100084, China

[5]Immunology Program, Sloan Kettering Institute, Memorial Sloan Kettering Cancer Center, New York, NY;

[6]Immunology and Microbial Pathogenesis Program, Weill Cornell Graduate School of Medical Sciences, Cornell University, New York, NY

Authorship Note: N. Yin and G. Jin contributed equally to this study

This PDF file includes:

Supplemental Figures 1 to 6
Legends for Supplemental Figures 1 to 6

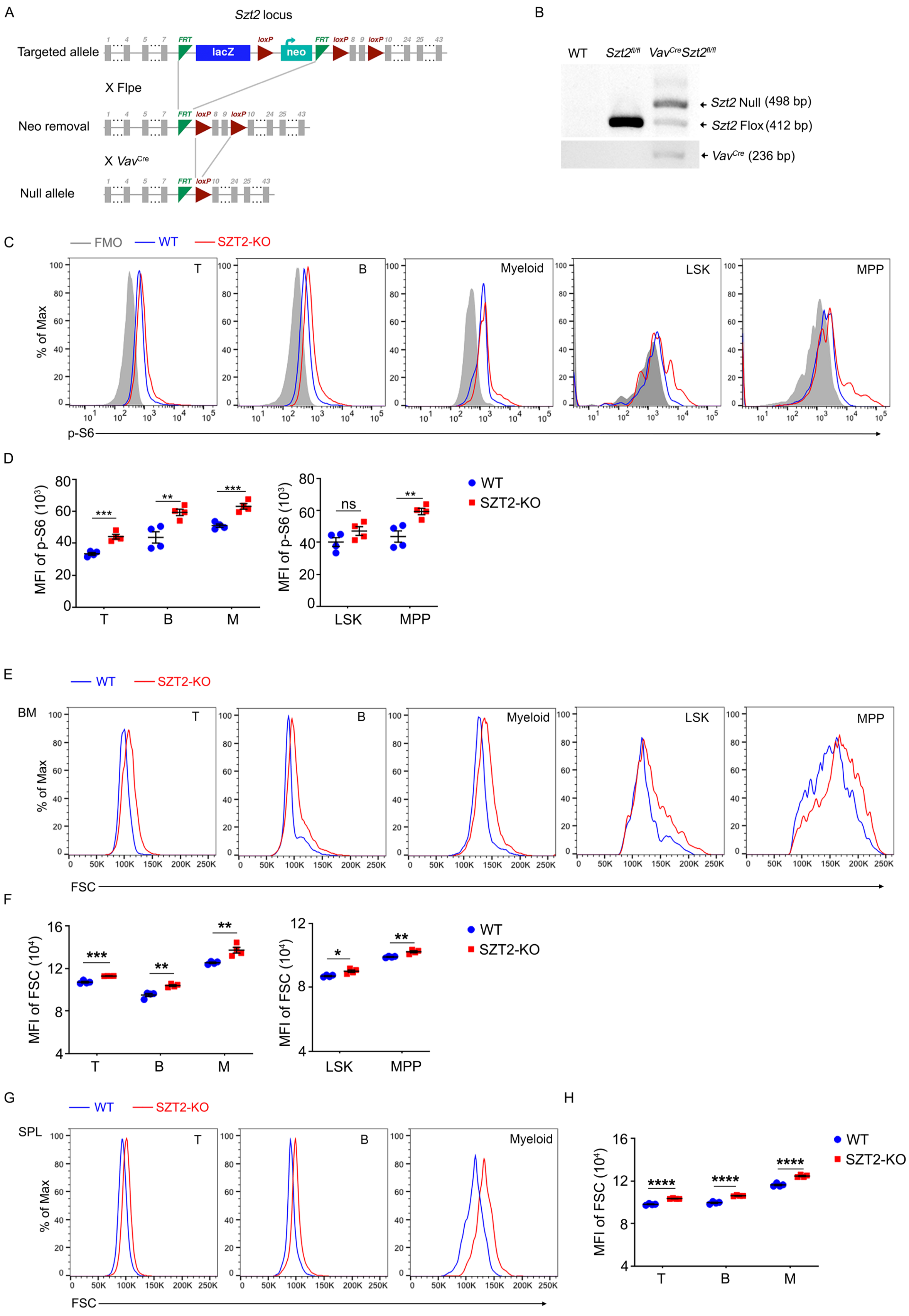

Supplemental Figure 1. SZT2 represses mTORC1 signaling in hematopoietic cells

(A) Targeting strategy of *Szt2* floxed mice. **(B)** PCR detection of floxed and null allele of *Szt2* in total BM cells. **(C** and **D)** Flow cytometry analysis of ribosomal S6 protein phosphorylation (pS6) of indicated populations in BM from WT and SZT2-KO mice at 14-week -old age. T cells ($CD3^+$), B cells ($B220^+$), myeloid cells ($CD11b^+$), LSK ($Lin^-Sca1^+c\text{-}Kit^+$) and MPP ($Lin^-Sca1^-c\text{-}Kit^+$). Representative plots (**C**) and statistics (**D**) are shown, n = 4 mice per genotype. Data shown are representative of 2 independent experiments. **(E** and **F)** Flow cytometry analysis of cell size of indicated populations in BM from 14-week-old WT and SZT2-KO mice. Representative plots (**E**) and statistics (**F**) are shown, n = 4 mice per group. Data shown are representative of 4 independent experiments. **(G** and **H)** Flow cytometry analysis of cell size of indicated populations in spleen (SPL) from 14-week-old WT and SZT2-KO mice. Representative plots (**G**) and statistics (**H**) are shown, n = 4 mice per genotype. Data shown are representative of 4 independent experiments. All error bars represent SEM, unpaired t-test, *$p < 0.05$, **$p < 0.01$, ***$p < 0.001$, ****$p < 0.0001$, ns, not significant.

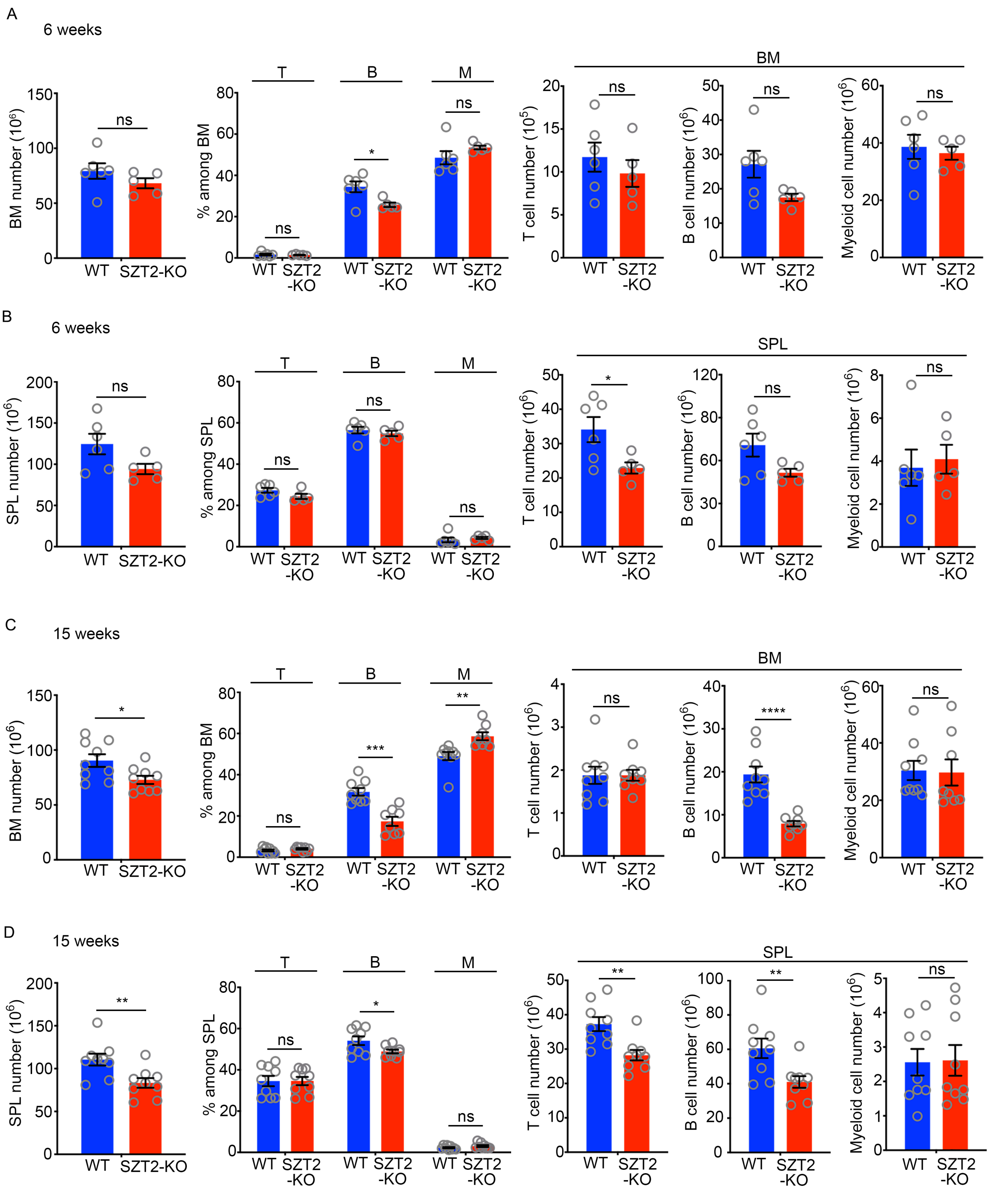

Supplemental Figure 2. Steady-state hematopoiesis is preserved in SZT2-KO mice

(A and B) Cell number and percentage of T cells ($CD3^+$), B cells ($B220^+$) and myeloid cells ($CD11b^+$) in bone marrow (BM) (**A**) and spleen (SPL) (**B**) from 6-week-old WT and SZT2-KO mice, n = 5 - 6 mice per genotype. Data shown are representative of 3 independent experiments. **(C and D)** Cell number and percentage of T cells, B cells and myeloid cells in

bone marrow (BM) (**C**) and spleen (SPL) (**D**) from 15-week-old WT and SZT2-KO mice, n = 8 - 9 mice per genotype. Data shown are representative of 2 independent experiments. All error bars represent SEM, unpaired t-test, *$p < 0.05$, **$p < 0.01$, ***$p < 0.001$, ****$p < 0.0001$, ns, not significant.

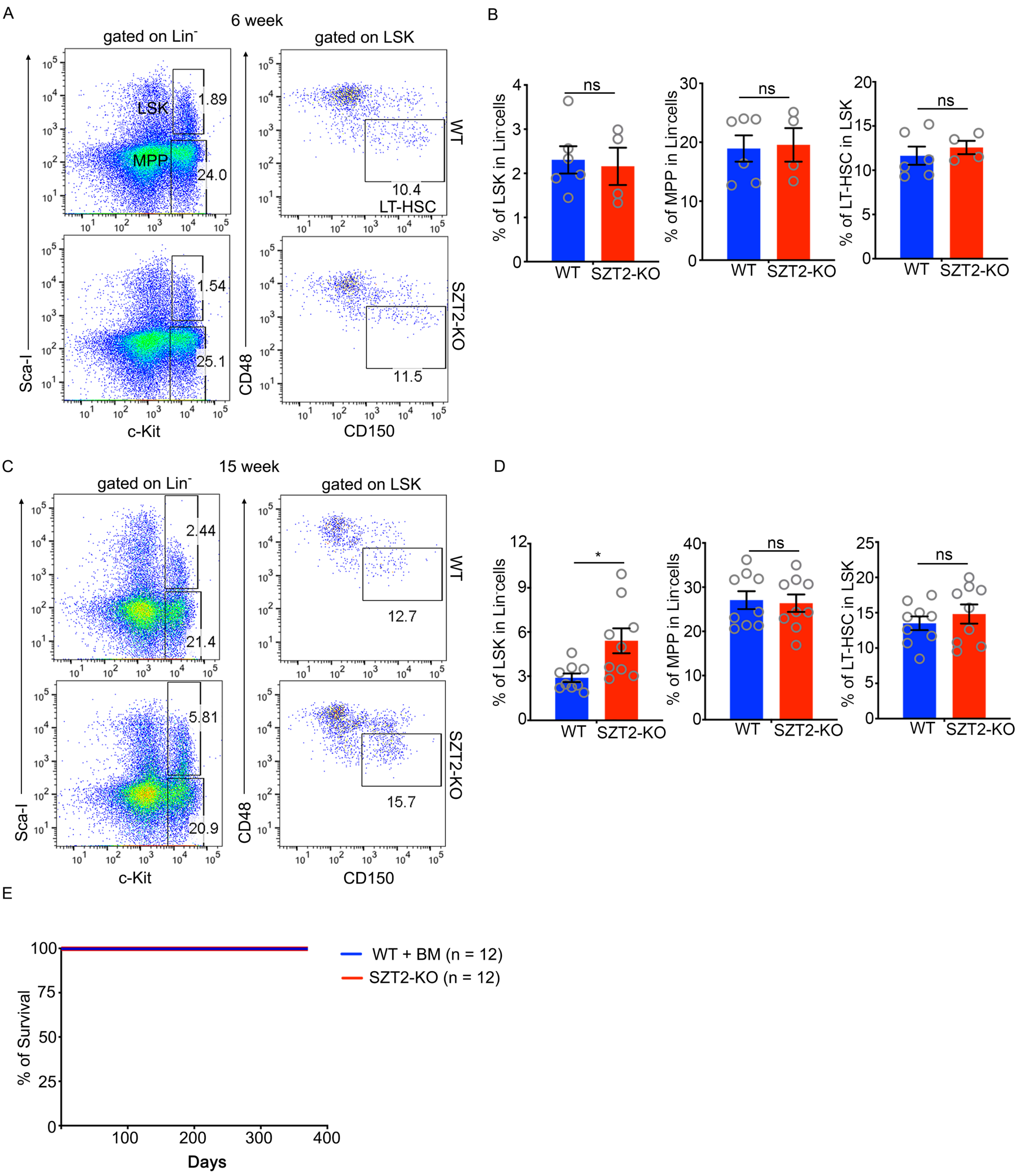

Supplemental Figure 3. HSC are preserved in SZT2-KO mice under steady-state

Flow cytometry analysis of LSK, MPP and LT-HSC populations in WT and SZT2-KO mice at different ages. (**A** and **B**) Representative plots (**A**) and statistics (**B**) from 6-week-old mice are shown, n = 4 - 6 mice per genotype. Data shown are representative of 3 independent experiments. (**C** and **D**) Representative plots (**C**) and statistics (**D**) from 15-week-old mice are shown, n = 9 mice per genotype. Data shown are representative of 2 independent experiments.

(**E**) Survival curves of WT and SZT2-KO mice. All error bars represent SEM, unpaired t-test, $*p < 0.05$, ns, not significant.

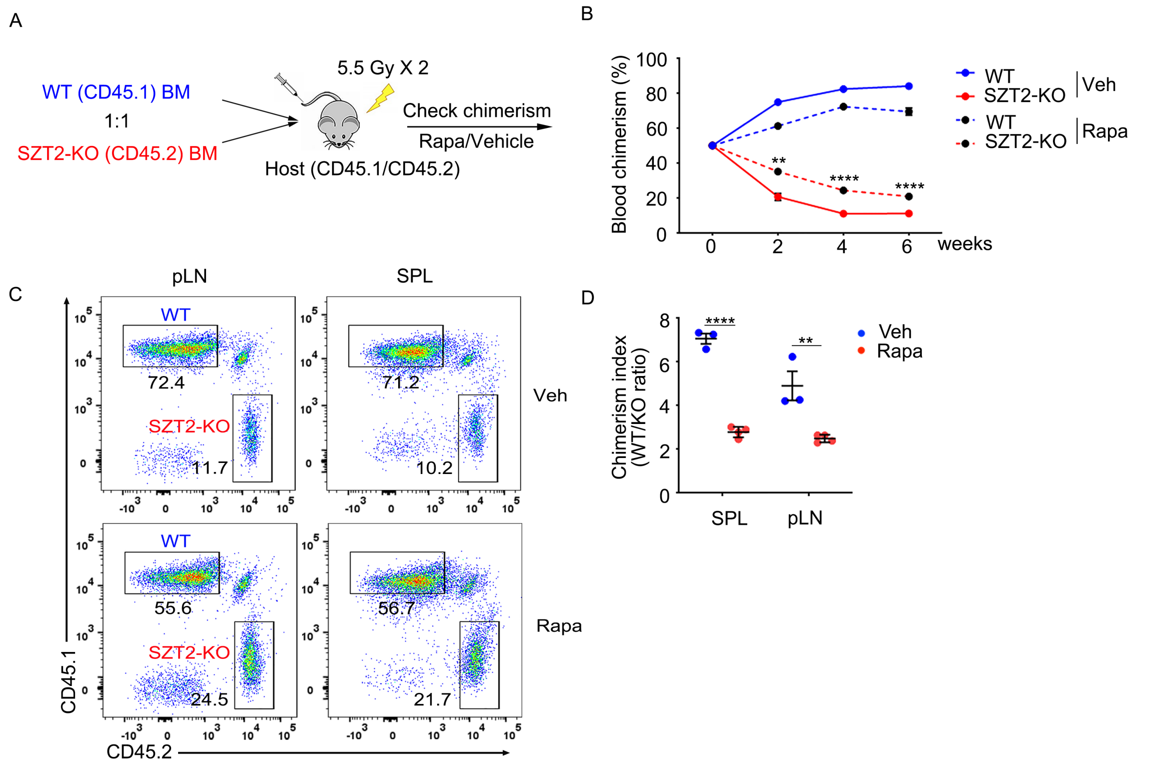

Supplemental Figure 4. Rapamycin treatment partially rescues the repopulating capacity of SZT2-KO HSC

(A) A diagram of BM chimera experiment. WT (CD45.1) and SZT2-KO (CD45.2) BM cells were mixed at 1:1 ratio and injected into lethally irradiated CD45.1/CD45.2 recipient mice, followed by intraperitoneal injection of rapamycin (Rapa) or vehicle (Vel) every 2 days for 6 weeks. Chimerism was monitored. **(B)** Blood chimerism of recipient mice at indicated time points. **(C and D)** Chimerism of peripheral lymph node (pLN) and spleen (SPL) at 6 weeks post-transplantation. Representative plots (**C**) and statistics data **(D)** are shown, n = 3 - 4 mice per genotype. Data shown are representative of 2 independent experiments. All error bars represent SEM, unpaired t-test, **p < 0.01, **** p< 0.0001.

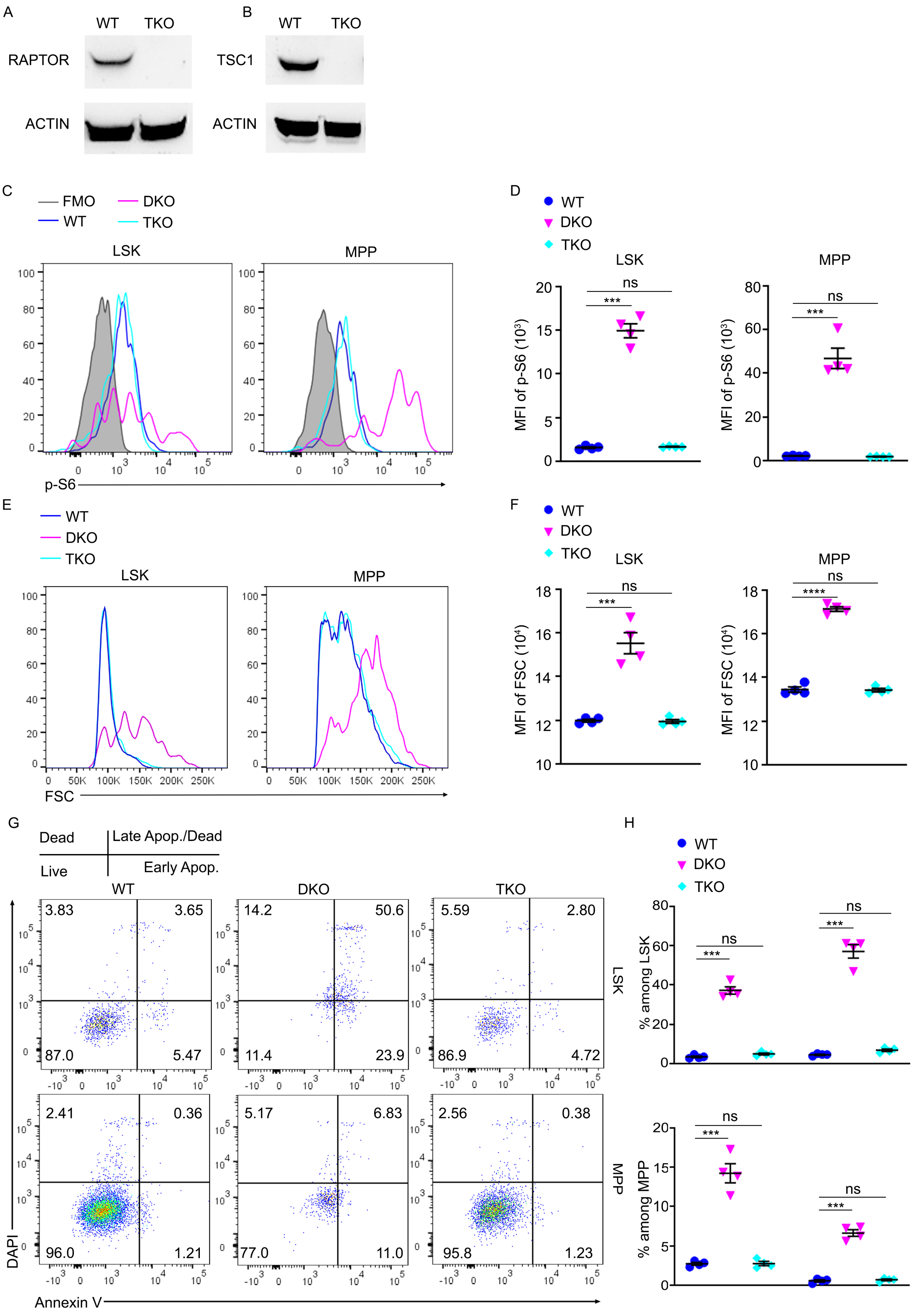

Supplemental Figure 5. Hyperactivation of mTOCR1 and increased ROS production in

HSC from DKO mice are reversed to wild-type level by RAPTOR-deficiency in TKO mice

(A and **B)** Immunoblot analysis of RAPTOR and TSC1 expression in bone marrow cells from WT and TKO mice. Data shown are representative of 2 independent experiments. **(C** and **D)** Phosphorylation of ribosomal S6 protein (pS6) in LSK and MPP population from WT, DKO or TKO mice at 4-week-old age was measured by flow cytometry. Representative plots **(C)** and statistics of mean fluorescence intensity (MFI) **(D)** are shown, n = 4 mice per genotype. Data shown are representative of 2 independent experiments. **(E** and **F)** Flow cytometry analysis of cell size of indicated populations in BM from 4-week-old mice. Representative plots **(E)** and statistics **(F)** are shown, n = 4 mice per genotype. Data shown are representative of 2 independent experiments. **(G** and **H)** Flow cytometry analysis of apoptosis of LSK and MPP population from mice with indicated genotypes at 4-week-old age. Representative plots **(G)** and statistics **(H)** are shown, n = 4 mice per genotype. Data shown are representative of 2 independent experiments. All error bars represent SEM, one-way ANOVA followed by Dunnett's test, *$p < 0.05$, *** $p< 0.001$, ns, not significant.

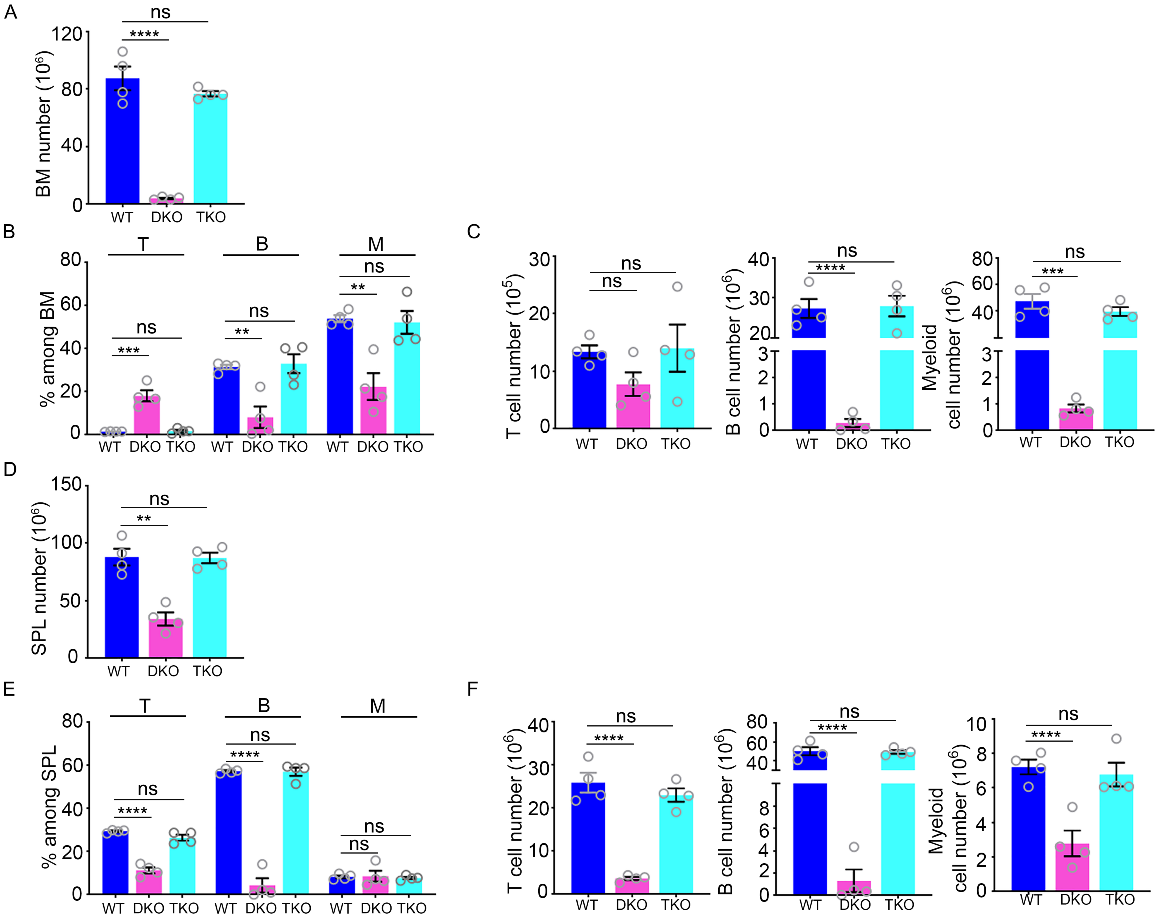

Supplemental Figure 6. Hematopoiesis defect of DKO mice is reversed to wild-type level by RAPTOR-deficiency in TKO mice

(A, **B** and **C)** Percentage and absolute number of T cells ($CD3^+$), B cells ($B220^+$) and myeloid cells ($CD11b^+$) in BM from 4-week-old WT, DKO or TKO mice, n = 4 mice per genotype. **(D, E** and **F)** Percentage and absolute number of T cells, B cells and myeloid cells in spleen (SPL) from mice with indicated genotypes, n = 4 mice per genotype. Data shown are representative of 2 independent experiments. All error bars represent SEM, one-way ANOVA followed by Dunnett's test, **p < 0.01, ***p < 0.001, ****p < 0.0001, ns, not significant.
